# LT-DeepLab: an improved DeepLabV3+ cross-scale segmentation algorithm for Zanthoxylum bungeanum Maxim leaf-trunk diseases in real-world environments

**DOI:** 10.3389/fpls.2024.1423238

**Published:** 2024-10-22

**Authors:** Tao Yang, Jingjing Wei, Yongjun Xiao, Shuyang Wang, Jingxuan Tan, Yupeng Niu, Xuliang Duan, Fei Pan, Haibo Pu

**Affiliations:** ^1^ College of Information Engineering, Sichuan Agricultural University, Ya’an, China; ^2^ School of Physics and Optoelectronic Engineering, Nanjing University of Information Science and Technology, Jiangsu, China; ^3^ Ya’an Digital Agricultural Engineering Technology Research Center, Sichuan Agricultural University, Ya’an, China

**Keywords:** Zanthoxylum bungeanum Maxim, cross-scale, real-world environments, disease segmentation, small target, deep learning, attention mechanism

## Abstract

**Introduction:**

Zanthoxylum bungeanum Maxim is an economically significant crop in Asia, but large-scale cultivation is often threatened by frequent diseases, leading to significant yield declines. Deep learning-based methods for crop disease recognition have emerged as a vital research area in agriculture.

**Methods:**

This paper presents a novel model, LT-DeepLab, for the semantic segmentation of leaf spot (folium macula), rust, frost damage (gelu damnum), and diseased leaves and trunks in complex field environments. The proposed model enhances DeepLabV3+ with an innovative Fission Depth Separable with CRCC Atrous Spatial Pyramid Pooling module, which reduces the structural parameters of Atrous Spatial Pyramid Pooling module and improves cross-scale extraction capability. Incorporating Criss-Cross Attention with the Convolutional Block Attention Module provides a complementary boost to channel feature extraction. Additionally, deformable convolution enhances low-dimensional features, and a Fully Convolutional Network auxiliary header is integrated to optimize the network and enhance model accuracy without increasing parameter count.

**Results:**

LT-DeepLab improves the mean Intersection over Union (mIoU) by 3.59%, the mean Pixel Accuracy (mPA) by 2.16%, and the Overall Accuracy (OA) by 0.94% compared to the baseline DeepLabV3+. It also reduces computational demands by 11.11% and decreases the parameter count by 16.82%.

**Discussion:**

These results indicate that LT-DeepLab demonstrates excellent disease segmentation capabilities in complex field environments while maintaining high computational efficiency, offering a promising solution for improving crop disease management efficiency.

## 1 Introduction

Zanthoxylum bungeanum Maxim, a woody plant in the Rutaceae family, is widely distributed across Asia. It serves as a significant economic resource, offering valuable seasoning, spice, and woody oilseed derivatives from its branches, leaves, and fruits. These parts also possess high nutritional and medicinal values (Lu et al., 2022). Under large-scale cultivation, the plant frequently suffers from diseases, particularly in its branches and leaves, significantly impacting yield. Early disease detection is crucial for preventing substantial economic losses in agriculture (Savary et al., 2019). Consequently, rapid and accurate monitoring and analysis of leaf and trunk diseases in Zanthoxylum bungeanum Maxim are essential. Traditional disease identification methods, which predominantly rely on subjective manual visual observation, are labor-intensive, slow, and prone to misclassification (Cruz et al., 2019).

In terms of traditional segmentation techniques, threshold-based methods are prevalent. Gao and Lin (2019) enhanced and extracted leaf veins to segment medicinal plant leaves using direct processing of RGB images and OTSU methods. Barbedo (2016) developed a semi-automatic algorithm for segmenting plant leaf disease symptoms by manipulating the histograms of the H-channel in HSV and the a-channel in Lab color space. Clustering-based approaches are also utilized; for instance, Shedthi et al. (2023) designed a plant disease recognition system using hybrid clustering algorithms to improve upon the local optimization limitations of the k-means algorithm. Javidan et al. (2023) employed new image processing algorithms and multi-class support vector machines for diagnosing and classifying grapevine leaf diseases, achieving up to 98.97% accuracy with PCA and GLCM feature selection. Additionally, there are region-based methods: Ma et al. (2017) proposed a segmentation method for vegetable leaf lesions using color information and region-growing techniques. They composed a comprehensive color feature using the red index, the H component in HSV color space, and the b component in Lab color space. Based on this feature, an interactive region-growing method was used to segment leaf lesions against a complex background. Li et al. (2018) developed a single-leaf segmentation method for indoor ornamental plant leaves using over-segmentation with small planes and region growing with small planes in a dense plant point cloud, achieving an average precision and recall rate exceeding 90%. These methods, while less computationally demanding and straightforward, often lack robustness in complex backgrounds due to subtle gray-scale variations and small diseased spot sizes on leaves.

With the continuous advancements in computer vision, high-performance models have been increasingly utilized for image classification, detection, and recognition tasks (Attri et al., 2023). There are currently three principal approaches for analyzing plant diseases using deep learning: image-based classification, bounding box-based object detection, and semantic segmentation based on pixel classification. Nahiduzzaman et al. (2023) developed a lightweight deep separable CNN model, PDS-CNN, achieving accuracies of 95.05% in triple classification and 96.06% in binary classification with a compact model size of 6.3M. Pal and Kumar (2023) combined traditional INC-VGGN and Kohonen-based networks for plant disease detection and severity classification. Thai et al. (2023) introduced FormerLeaf for cassava leaf disease detection, employing the Least Important Attention Pruning (LelAP) algorithm to enhance Transformer models by reducing model size by 28% and improving accuracy by approximately 3%. Additionally, they utilized the sparse matrix multiplication method (SPMM) to decrease the model’s complexity, reducing training time by 10%. Liu et al. (2024) proposed Fusion Transformer YOLO, a real-time and lightweight detection model that integrates VoVNet into the backbone to enhance accuracy and incorporates an improved dual-stream PAN+FPN structure in the neck, achieving an average model accuracy of 90.67%. Jodas et al. (2021) merged deep residual blocks with UNet for semantic segmentation, achieving an IoU of 81.47% by refining the segmentation region to exclude irrelevant binary areas. Zhang et al. (2023) improved the sensory field in a grapevine leaf disease segmentation model by inverting the residual convolution and replacing the downsampling operation with reversible attention, increasing IoU performance by 4.04% over the baseline model. Compared to traditional methods, semantic segmentation offers more practical and complex functionalities, making it highly suitable for precision agriculture applications (Deng et al., 2023).

Unlike previous studies, our task requires cross-scale segmentation due to varying sizes of diseased trunks and frost-damaged parts, which differ from the smaller diseased leaves and spots. The ASPP structure of Deeplabv3+ is particularly apt for cross-scale feature extraction due to its varied receptive fields, making it an ideal baseline for our study on Zanthoxylum bungeanum Maxim trunk and leaf disease segmentation. In our experiments, we identified two main challenges: (1) significant loss of target edge information in complex backgrounds, leading to poor segmentation under varied environmental conditions and blurred target boundaries, and (2) the difficulty in detecting and segmenting small disease spots on leaves due to their irregular size and presence.

To address the issue of complex backgrounds, Wang et al. (2021) fused DeepLabV3 and UNet in a two-stage model for cucumber leaf lesion segmentation, initially segmenting leaves in complex backgrounds with DeepLabV3 followed by lesion segmentation with UNet. Mzoughi and Yahiaoui (2023) segmented diseases based on local disease signature features, reducing the impact of common backgrounds. To mitigate computational costs, this paper designs a lightweight dual-attention mechanism that concurrently extracts features from both channel and spatial dimensions, focusing the model on target regions while disregarding background noise.

To tackle the problem of overlooking small leaf spots, Qi and Jia (2023) enhanced segmentation accuracy for small infrared targets by modifying the expansion rate of the ASPP module and introducing a position enhancement module. Deng et al. (2023) developed a cross-layer attention fusion mechanism to differentiate tiny spots from healthy areas. This paper enhanced the ASPP module by altering its data flow, adding deformable convolution, and incorporating our proposed CRCC module to better detect small target spots. Additionally, standard convolution is replaced with depth-separable convolution to reduce parameter count while improving accuracy. Furthermore, deformable convolution is applied to shallow extracted features before their integration with deep features to more effectively transfer shallow information.

In this paper, a cross-scale disease segmentation network is proposed, LT-DeepLab, for Zanthoxylum bungeanum Maxim trunks and leaves. The contributions of this study are summarized as follows: (1) A dual-attention module CRCC structure is designed, combining spatial and channel attention mechanisms to enhance segmentation in complex backgrounds. (2) An improved ASPP module (FDCASPP) is proposed, incorporating an enhanced attention mechanism with variability convolution to boost cross-scale feature extraction and using lightweight deep separable convolution to minimize redundant information. (3) The model employs a deep supervision technique that does not increase parametric quantities and incorporates an auxiliary loss during training to enhance accuracy. (4) This paper innovatively applies semantic segmentation techniques to Zanthoxylum bungeanum Maxim disease segmentation, producing a scientific dataset of Zanthoxylum bungeanum Maxim leaf and trunk disease and facilitating cross-scale segmentation of leaf and trunk diseases, thereby bridging the research gap in this area.

## 2 Materials and methods

### 2.1 Data collection and processing

#### 2.1.1 Data collection

**Figure 1:**
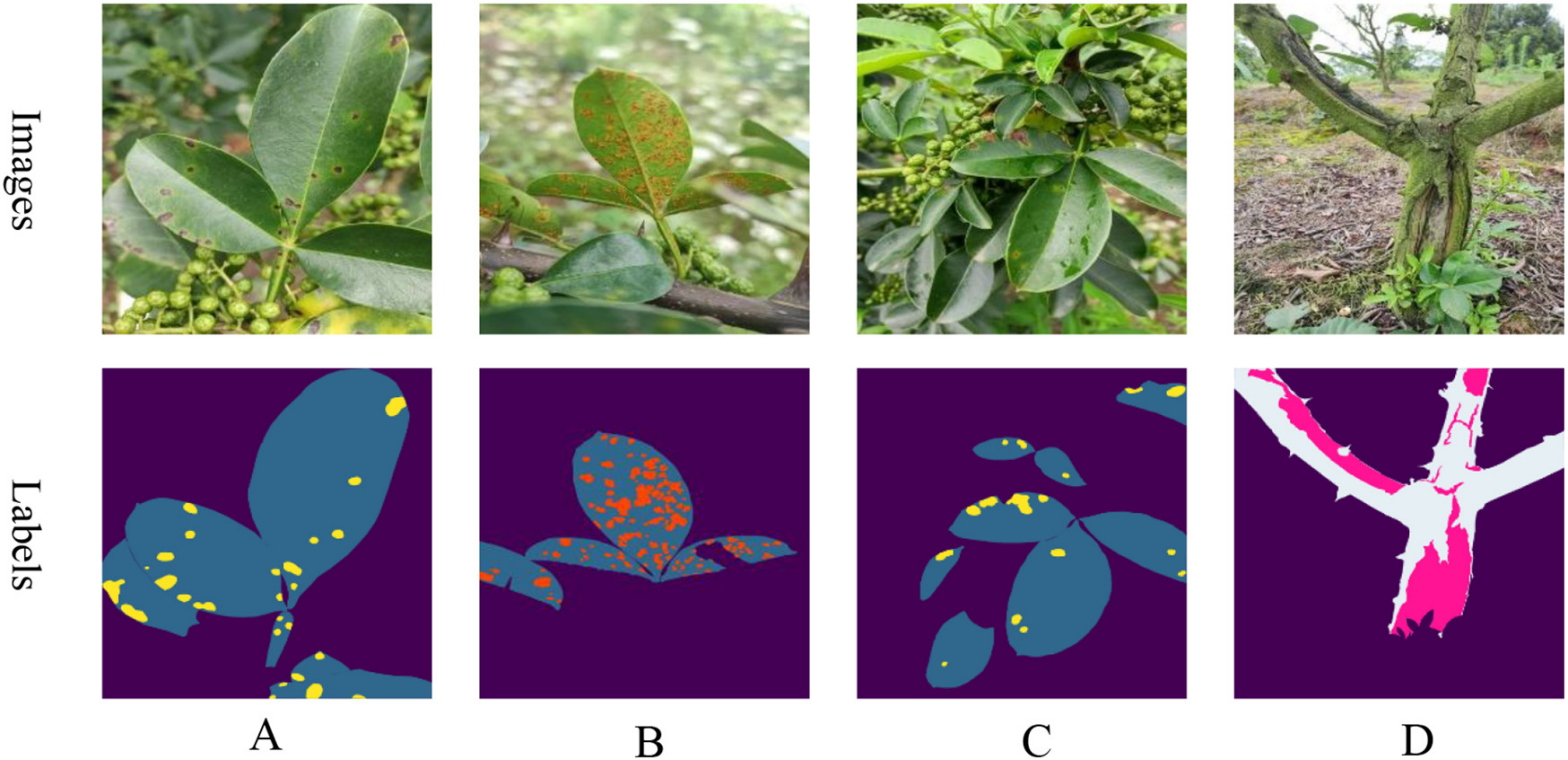
Samples of various diseases: **(A)** leaf spot; **(B)** rust; **(C)** postrainy leaf spot; **(D)** frost damage.

This study examines the segmentation of diseases on the leaves and trunks of Zanthoxylum bungeanum Maxim trees within complex environments. The image data were collected from a Zanthoxylum bungeanum Maxim plantation located in Dongba Town, Nanbu County, Sichuan Province, China. To accommodate diverse lighting conditions in natural settings, the dataset was compiled at various times in July, specifically in the morning (8:00-10:00), at noon (12:00-14:00), and in the afternoon (15:00-17:00), with additional images captured post-rainfall. The categories of images include leaf spot, rust, and frost damage. Representative examples of these images are presented in **Figure 1**.

#### 2.1.2 Data processing

A total of 1,200 raw images were captured with an initial resolution of 3472x4624. Leaf and disease spots, as well as trunk and frost damage, were annotated using Labelme software under expert guidance to create mask maps. The dataset was then split into a training set of 960 images and a test set of 240 images, adhering to an 8:2 ratio. The annotated images are depicted as labels in **Figure 1**.Since the initial training set comprised only 960 images, this paper applies data augmentation to boost the model’s robustness and generalization capabilities. To maintain a reasonable training speed, the augmentation process involved randomly scaling the length of the images to a range between 2048 and 512 pixels, then cropping them to a size of 512x512 pixels, and finally applying random flips. Additionally, transformations in terms of brightness, contrast, and saturation were used to further improve the model’s performance. These data augmentation techniques were applied consistently across all experiments to ensure uniformity among the different models.

### 2.2 Improved methods

Based on DeepLabv3+, this paper proposes a segmentation network named LT-DeepLab designed for cross-dimensional segmentation of Zanthoxylum bungeanum Maxim trunks, leaves, and lesions. The network primarily consists of deformable convolutions, a fission feature pyramid with depth-separable convolutions, and an improved CRCC dual attention module. The CRCC module combines the Criss-Cross module with the Convolutional Block Attention Module, allowing for feature complementation in both spatial and channel dimensions, and is used in both the backbone and FDCASPP modules. Furthermore, the FDCASPP module incorporates deformable convolutions and depth-separable convolutions, reducing the parameter count while maintaining or even improving model accuracy.

#### 2.2.1 DeepLabv3+ network structure

DeepLabv3+ is a prominent semantic segmentation architecture distinguished by its Atrous Spatial Pyramid Pooling (ASPP) module, which employs dilated convolution to capture contextual information across various scales (Chen et al., 2018). This is achieved by applying differing dilation rates to feature maps processed by deep neural networks, which are then combined with low-level features to produce the prediction map. However, the original model had a high parameter count and did not perform well in segmentation for this specific task, prompting us to make several improvements.

#### 2.2.2 LT-DeepLab structure

**Figure 2:**
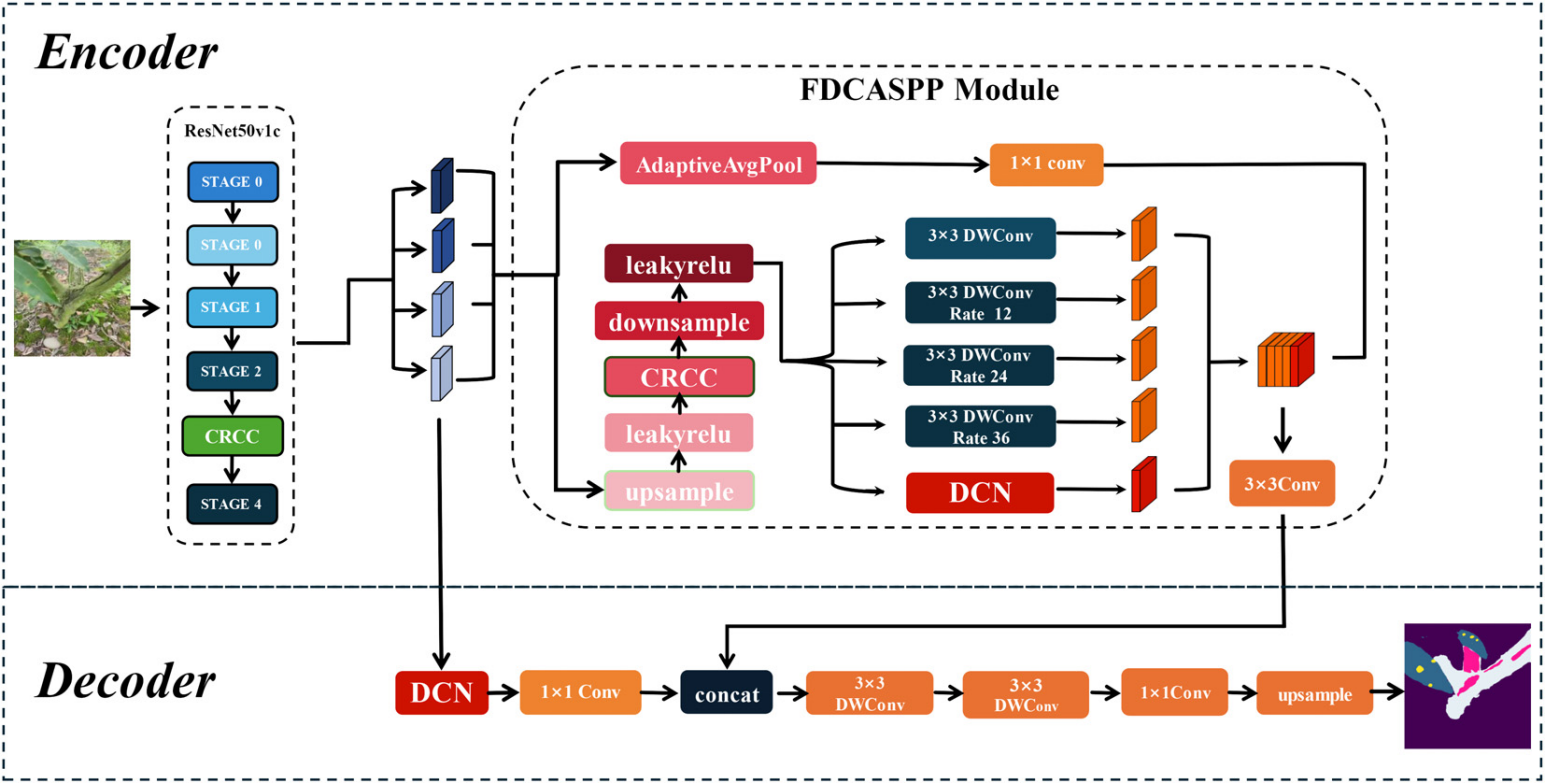
LT-DeepLab network structure.

In real environments, the segmentation of leaf and trunk diseases is complicated by various factors such as light, weather, shading, and complex backgrounds, particularly when imaging leaf spots and frost-damaged trunk portions. This study addresses both the larger-sized trunk and frost-damaged parts as well as the smaller leaves and even smaller diseased spots, with the inherent data imbalance increasing segmentation difficulty. Although the traditional DeepLabv3+ network, with its ASPP module capable of multi-scale feature extraction, achieves satisfactory segmentation results on leaves and trunks against a single background, it struggles with more complex backgrounds. The performance deteriorates further due to the cross-pixel feature extraction of the expansion convolution within the ASPP module, often failing to adequately capture features of leaves and smaller spots, which is critical for segmentation tasks involving small targets (Zhu et al., 2023). To address these challenges, this study introduces an enhanced version of DeepLabv3+, LT-DeepLab, as depicted in **Figure 2**. This model integrates an improved Criss-Cross attention mechanism to boost the feature extraction capability of the backbone. In the decoder, features from the backbone are fused with outputs from the ASPP module, incorporating deformable convolution to better preserve features of small targets like leaves and disease spots. Furthermore, this paper proposes a new encoder, the Separation Fission Depth Separable with CRCC Atrous Spatial Pyramid Pooling, called FDCASPP module. This encoder replaces standard convolution with depth-separable convolution, retaining the multi-scale feature extraction of ASPP while integrating an enhanced CRCC module and deformable convolution, thus addressing the insufficient feature extraction capability for small targets.

#### 2.2.3 CRCC module structure

In real-world scenarios, the segmentation of Zanthoxylum bungeanum Maxim leaves and trunks is challenged by frequent occlusions and the phenotypic similarity between healthy and diseased leaves. To improve the model’s focus on relevant features and enhance information filtration from complex backgrounds, this paper introduces and refines the Criss-Cross attention module (Huang et al., 2019). The enhanced CCNet efficiently captures contextual information from the surrounding pixels via cross-path operations. This mechanism enables each pixel to ascertain the remote dependencies of all other pixels through a cyclic operation, thereby improving segmentation accuracy. The original Criss-Cross module is computed as, given a local feature map $H\in {\mathbb{R}}^{C\times W\times H}$ , the feature maps K, Q, and V are first generated for the leaf and trunk lesion feature maps after three 1 × 1 convolutions, where { Q,K } $\in {\mathbb{R}}^{C^{\prime }\times W\times H}$ , and $C^{\prime }$ is the number of channels less than C . After that, this paper generates the feature maps Q and K by **Affinity** operation to generate the attention map $A\in {\mathbb{R}}^{\left(H+W-1\right)\times W\times H}$ , and for the position u of the feature map Q in the spatial dimension, which can obtain $Q_{u}\in {\mathbb{R}}^{C^{\prime }}$ , and for the same row or column of the same position u in K , it can obtain $\Omega_{u}\in {\mathbb{R}}^{\left(H+W-1\right)\times C^{'}}$ . $\Omega_{i,u}\in {\mathbb{R}}^{C^{\prime }}$ is the ith element of $\Omega_{u}$ . Define the **Affinity** operation as follows:


(1) $$\begin{array}{c} d_{i,u}=Q_{u}{\text{Ω}}_{i,u} \end{array}$$

Where $d_{i,u}\in D$ is the degree of correlation between feature $Q_{u}$ and $\Omega_{i,u}$ , $i=\left[1,\ldots ,\left|\Omega_{u}\right|\right]$ , $D\in {\mathbb{R}}^{\left(H+W-1\right)\times W\times H}$ . Then, a softmax layer on D over the channel dimension is applied to calculate the attention map A .

Correspondingly, for the previously generated feature map $V\in {\mathbb{R}}^{C\times W\times H}$ and each position u in the spatial dimension, $V_{u}\in {\mathbb{R}}^{C}$ and a set $\Phi_{u}\in {\mathbb{R}}^{\left(H+W-1\right)\times C}$ are obtained, where the set $\Phi_{u}$ is the set of feature vectors in V that are in the same row or column as position u . **Aggregation** is defined as:


(2) $$\begin{array}{c} H_{u}^{'}=\sum_{i\in \left|{\text{Φ}}_{u}\right|}A_{i,u}{\text{Φ}}_{i,u}+H_{u} \end{array}$$

where $\;H_{u}^{'}$ is the output feature maps $H^{\prime }\in {\mathbb{R}}^{C\times W\times H}$ at position u . $A_{i,u}$ is the scalar value at a for channel i and position u .

**Figure 3:**
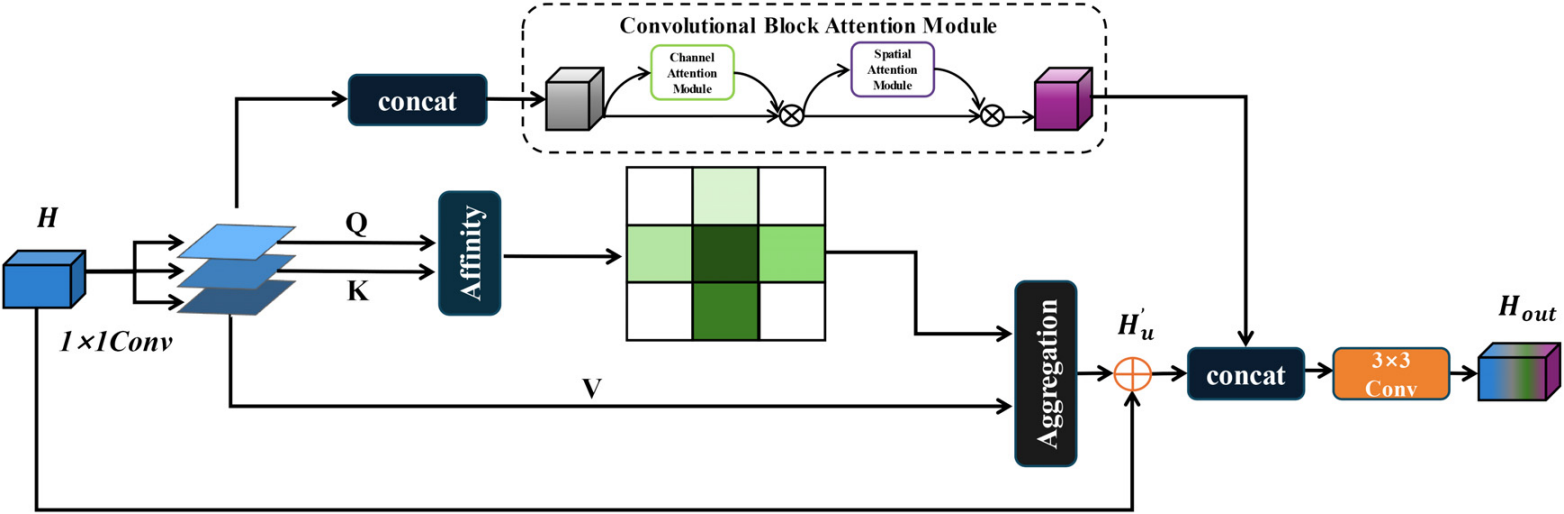
CRCC module structure.


However, while the Criss-Cross Attention module effectively contextualizes features spatially, it does not adequately connect spatial information (Huang et al., 2019). To address this limitation, this paper integrated it with the Convolutional Block Attention Module (CBAM) (Woo et al., 2018), creating a dual attention mechanism named CRCC, as illustrated in **Figure 3**. In this mechanism, the feature maps K, Q, and V from H are fused before being processed by the CBAM module, which then weights these features to ensure a cohesive channel connection. The integration process is detailed as follows:

(3) $$\begin{array}{c} H_{c}=CBAM\left(concat\left(K,Q,V\right)\right) \end{array}$$

Finally, the output of the CRCC module is combined with that of the Criss-Cross module. To capture the global connections in a cyclic manner, a two-dimensional convolution is applied to integrate the features and compress their dimensionality. This process preserves the original spatial context provided by the Criss-Cross module while incorporating the spatial and channel-weighted features from the CBAM. The final output of the CRCC module after one iteration is described below:


(4) $$\begin{array}{c} H_{out}=Conv2d\left(H_{c}+H_{u}^{'}\right) \end{array}$$

Additionally, this paper has integrated the CRCC module into the enhanced Fission Depth Separable with CRCC Atrous Spatial Pyramid Pooling (FDCASPP) structure to further optimize our segmentation network.

#### 2.2.4 Deformable convolution structures

**Figure 4:**
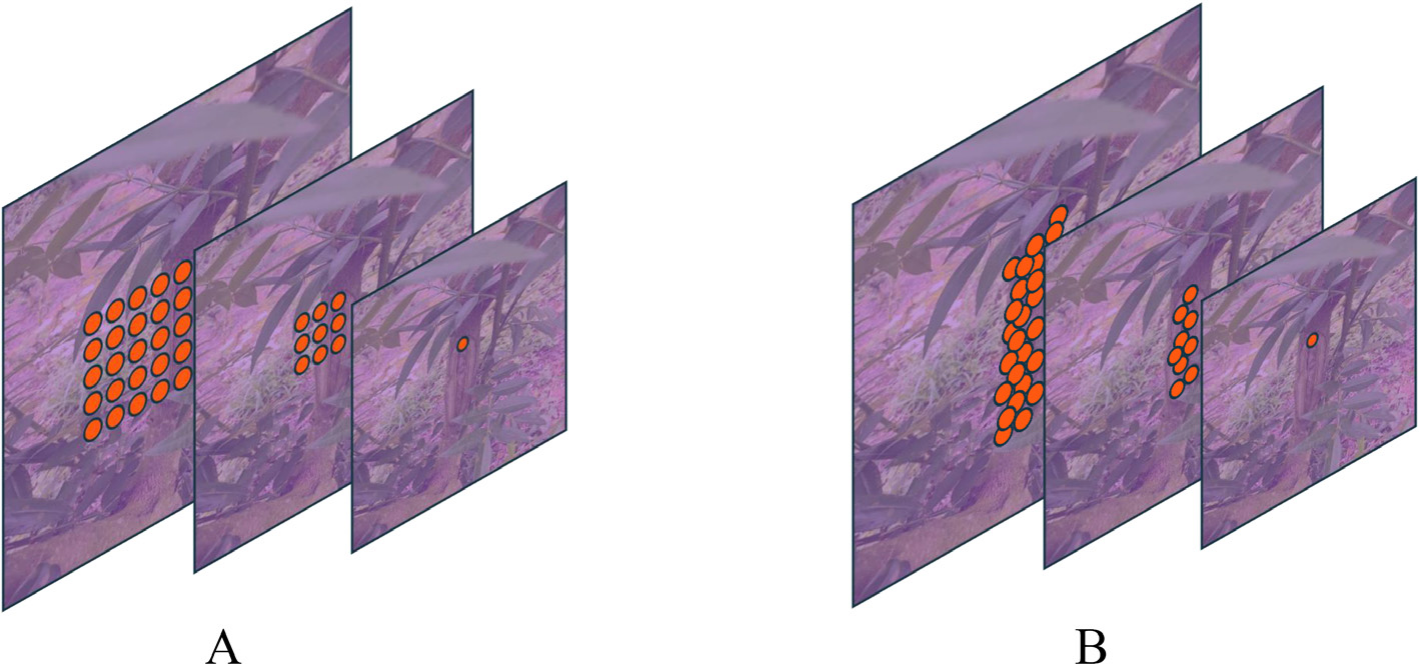
Comparison of conventional and deformable convolution receptive fields: Panel **(A)** displays a schematic diagram of the receptive field for a standard 3x3 convolution, while Panel **(B)** illustrates the receptive field of deformable convolution.


Conventional convolution operates with a fixed kernel shape, which may not adequately address the irregular shapes of leaf and trunk lesions. To overcome this limitation, this paper introduces deformable convolution, which modifies the convolution process to adapt to these irregularities before feature fusion. In standard 2D convolution, feature maps for leaf and trunk lesions are initially sampled using a regular grid network R . The sampled values are then multiplied by their corresponding weights w , and subsequently summed. The output at each position $P_{0}$ on the feature maps y , can be described by the following equation (Dai et al., 2017):


(5) $$\begin{array}{c} y\left(P_{0}\right)=\sum_{P_{n}\in R}W\left(P_{n}\right)\cdot X\left(P_{0}+P_{n}\right) \end{array}$$

where $P_{n}$ represents each position on the convolution kernel. As illustrated in **Figure 4**, deformable convolution modifies standard convolution by introducing an offset to the receptive field. Consequently, Equation 5 transforms into Equation 6. This offset is learnable, allowing it to adapt closely to the actual contours of the object. Through ablation studies, deformable convolution has demonstrated enhanced segmentation capabilities, significantly improving the model’s performance.

(6) $$\begin{array}{c} y\left(P_{0}\right)=\sum_{P_{n}\in R}W\left(P_{n}\right)\cdot X\left(P_{0}+P_{n}+\text{Δ}P_{n}\right) \end{array}$$

#### 2.2.5 FDCASPP module structure

The traditional Atrous Spatial Pyramid Pooling module utilizes specific expansion rates to obtain different receptive fields for multi-scale feature extraction. However, due to the integration of pooling and convolution with strides, there is significant loss of boundary information in the segmented targets. Additionally, the extensive use of expansive convolutions through a deep convolutional neural network with a high number of channels results in a large parameter count. To address these issues, this paper proposes a Fission Depth Separable with CRCC Atrous Spatial Pyramid Pooling (FDCASPP). This module divides the feature maps into two data streams: one stream undergoes global average pooling followed by a 1x1 convolution for global feature statistics, while the other stream reduces the feature map resolution to balance accuracy with computation. As demonstrated by Xu et al. (2015), LeakyReLU outperforms ReLU in scenarios involving small datasets. To enhance model expressiveness, the reduced feature map is activated using LeakyReLU, then processed through the CRCC dual attention mechanism, and subsequently enhanced for activation. It is then integrated into the multidimensional joint feature extraction section, which replaces the standard convolution in the original ASPP module with depth-separable convolution (Sifre and Mallat, 2014) to minimize redundant parameters. This section sets expansion rates at 12, 24, and 36 to accommodate various target sizes. Additionally, deformable convolution is employed to refine the segmentation of targets. Finally, the feature maps from both data streams in the FDCASPP are fused to enhance feature integration.

#### 2.2.6 Auxiliary head loss

To optimize the training process, Zhao et al. (2017) demonstrated in their study on PSPNet that employing auxiliary loss can significantly enhance training effectiveness. They established that setting the weight α of the auxiliary loss to 0.4 is optimal. Notably, the auxiliary head, which processes the feature maps from the backbone network to generate segmentation masks and calculate the auxiliary loss using the cross-entropy loss function, is active only during the training phase. Consequently, it does not add to the computational load or the parameter count during model inference. This paper adopts a similar approach by introducing auxiliary loss generated by the FCN auxiliary head, applying a cross-entropy function, with the weight also set to α=0.4.

### 2.3 Model training

The hardware configurations for training and testing in this study include an 18 vCPU AMD EPYC 9754 128-Core Processor with 60GB of RAM and an NVIDIA RTX 3090 GPU with 24GB of video memory. The software environment consists of CUDA version 11.1, PyTorch version 1.8.1, and Python version 3.8.10. To mitigate the influence of hyper-parameters on experimental outcomes, this paper standardizes settings across all tests. Specifically, the Stochastic Gradient Descent optimizer was employed with an initial learning rate of 0.01. A polynomial decay strategy, PolyLR, was used to adjust the learning rate during the experiments. The experiments were conducted over 10,000 iterations with a batch size of 4.

### 2.4 Evaluation metrics

In this study, the effectiveness of disease segmentation on Zanthoxylum bungeum Maxim leaves and trunks is quantitatively assessed using three principal evaluation metrics: mean Intersection over Union (mIoU), mean Pixel Accuracy (mPA), and Overall Accuracy (OA). These metrics are chosen to provide a comprehensive evaluation of the segmentation performance across all tested networks. mIoU, mPA, and OA were formulae are calculated as follows, respectively:


(7) $$\begin{array}{c} mIoU=\frac{1}{k+1}\sum_{i=0}^{k}\frac{P_{ii}}{\sum_{j=0}^{k}P_{ij}+\sum_{j=0}^{k}P_{ji}-P_{ii}} \end{array}$$

(8) $$\begin{array}{c} mPA=\frac{1}{k+1}\sum_{i=0}^{k}\frac{P_{ii}}{\sum_{j=0}^{k}P_{ij}} \end{array}$$

(9) $$\begin{array}{c} OA=\frac{\sum_{i=0}^{k}P_{ii}}{\sum_{i=0}^{k}\sum_{j=0}^{k}P_{ij}} \end{array}$$

where k denotes the number of classes, excluding background $P_{ij}$ denotes the number of pixels that refer to the prediction of category i  as category j For the number of parameters of the model and the amount of computation, it is calculated as:


(10) $$\begin{array}{c} Parameters=C_{in}\times C_{out}\times K\times K \end{array}$$

(11) $$\begin{array}{c} \text{FLOPs}\;=C_{out}\times \left(C_{in}\;\times K^{2}\right)\times W\times H \end{array}$$

Where $C_{in}$ ​ denotes the number of input channels, $C_{out}$ ​ represents the number of output channels, K refers to the size of the convolutional kernel, and W and H indicate the width and height of the feature map, respectively.

Equation (10) describes how the number of parameters is calculated in each convolutional layer, the smaller the number of parameters is calculated, the lighter the model is and the easier it is to deploy. Equation (11) describes how the amount of computation in each convolutional layer is calculated, the smaller the amount of computation in the model, the smaller the computational burden of the model and the faster the inference. The bias terms in the convolutional layers are not considered in either of the above calculations.

### 2.5 Normalization of the confusion matrix

In this study, we utilized confusion matrices to evaluate the performance of the baseline model and our proposed model on the test set. To provide a more intuitive understanding of each category’s performance, we applied row normalization to the original confusion matrix. This process converts the absolute counts into proportions, indicating the percentage of samples within each category that are predicted to belong to respective categories. Specifically, each element in the normalized confusion matrix, denoted as $CM_{norm}$ , can be expressed as:


(12) $$\begin{array}{c} CM_{norm}\left[i,j\right]=\frac{CM\left[i,j\right]}{\sum_{k=1}^{N}CM\left[i,k\right]} \end{array}$$

Where CM[i,j] represents the element in the i th row and j th column of the original confusion matrix, indicating the number of samples from the actual category i that are predicted as category j , and $\sum_{k=1}^{N}CM\left[i,k\right]\;$ is the sum of all elements in the i th row, representing the total number of samples in the actual category i .

### 2.6 Statistical testing method

In this study, we employed a t-test to compare the performance differences between the improved LT-DeepLab model and the baseline model. To facilitate a statistical comparison, we recorded the results of five experiments conducted on the same dataset for both models. We utilized an independent samples t-test to assess whether the mean difference between these two models is statistically significant. Specifically, we calculated the means, variances, and t-statistics for the two samples, and determined the p-value by consulting the t-distribution table. The p-value represents the probability of observing the current t-statistic, or a more extreme value, under the null hypothesis that there is no significant difference between the means of the two groups. If the calculated p-value is less than the predetermined significance level (e.g., 0.05), we reject the null hypothesis, indicating that the mean difference between the two datasets is statistically significant. The calculation method is as follows:


(13) $$\begin{array}{c} \bar{X}=\frac{1}{n}\sum_{i=1}^{n}X_{i} \end{array}$$

(14) $$\begin{array}{c} S^{2}=\frac{1}{n-1}\sum_{i=1}^{n}{\left(X_{i}-\bar{X}\right)}^{2} \end{array}$$

(15) $$\begin{array}{c} t=\frac{\left|\bar{X_{1}}-\bar{X_{2}}\right|}{\sqrt{\frac{S_{1}^{2}}{n_{1}}+\frac{S_{2}^{2}}{n_{2}}}} \end{array}$$

(16) $$\begin{array}{c} df=\frac{{\left(\frac{S_{1}^{2}}{n_{1}}+\frac{S_{2}^{2}}{n_{2}}\right)}^{2}}{\frac{{\left(\frac{S_{1}^{2}}{n_{1}}\right)}^{2}}{n_{1}-1}+\frac{{\left(\frac{S_{2}^{2}}{n_{2}}\right)}^{2}}{n_{2}-1}} \end{array}$$

In this context, $\bar{X}\;$ represents the sample mean, with $\bar{X_{1}}$ ​ and $\bar{X_{2}}$ ​ denoting the means of the two groups. $S^{2}$ denotes the sample variance, with $S_{1}^{2}$ ​ and $S_{2}^{2}$ ​ representing the variances of the two groups. n stands for the sample size, with $n_{1}$ ​ and $n_{2}$ ​ indicating the sample sizes of the two groups. The t-statistic t measures the difference between sample means relative to the variability within the samples, offering a standardized metric of the mean difference. The degrees of freedom df are used to consult the t-distribution table to determine the p-value.

## 3 Experiments and analysis of results

### 3.1 Comparison experiments

To establish the superior segmentation capabilities of LT-DeepLab, this paper performes comparative experiments using an identical dataset across various state-of-the-art semantic segmentation networks. Each competing CNN-based model utilized a ResNet50V1c backbone, was subjected to the same data augmentation techniques, and employed a transfer learning approach. All networks were initialized with pre-trained weights from the Cityscapes dataset. The comparative analysis included models such as FCN, CCNet, DANet, PSPNet, Non_Local, UNet, and Segformer. The outcomes of these experiments are detailed in **Table 1**. As illustrated in **Table 1**, among the nine networks compared, our network, LT-DeepLab, consistently achieves the best results across all metrics, underscoring its distinct effectiveness for this task. Specifically, LT-DeepLab shows improvements over the baseline network by 3.59% in mIoU, 2.49% in mPA, and 0.63% in OA. Segformer, incorporating the advanced Transformer architecture, ranks second but still trails by 2.73%, 2.16%, and 0.94% in mIoU, mPA, and OA, respectively. Further comparisons reveal that our network surpasses CCNet, which utilizes the original Criss-Cross module. Our enhanced Criss-Cross attention module improves performance in mIoU, mPA, and OA by substantial margins of 3.23%, 1.54%, and 0.78%, respectively. Additionally, the inclusion of an auxiliary FCN head in our architecture enables it to outperform the native FCN network by 3.43% in mIoU, 2.62% in mPA, and 0.69% in OA. Against the classical PSPNet and DANet, LT-DeepLab also shows superior performance, leading by 3.28%, 2.79%, 0.50% and 2.90%, 2.01%, 0.52% in mIoU, mPA, and OA, respectively.

**Table 1:** Comparison of common semantic segmentation networks.

Model	mIoU	mPA	OA
DeepLabV3+(baseline)	72.99	83.53	95.36
FCN	73.15	83.40	95.30
CCNet	73.35	84.48	95.21
DANet	73.68	84.01	95.47
PSPNet	73.30	83.23	95.49
Non_Local	73.11	83.93	95.02
Segformer	73.85	83.86	95.05
UNet	70.42	81.70	93.02
**LT-DeepLab**	**76.58**	**86.02**	**95.99**

Bold indicates that this metric has the best performance.

**Figure 5:**
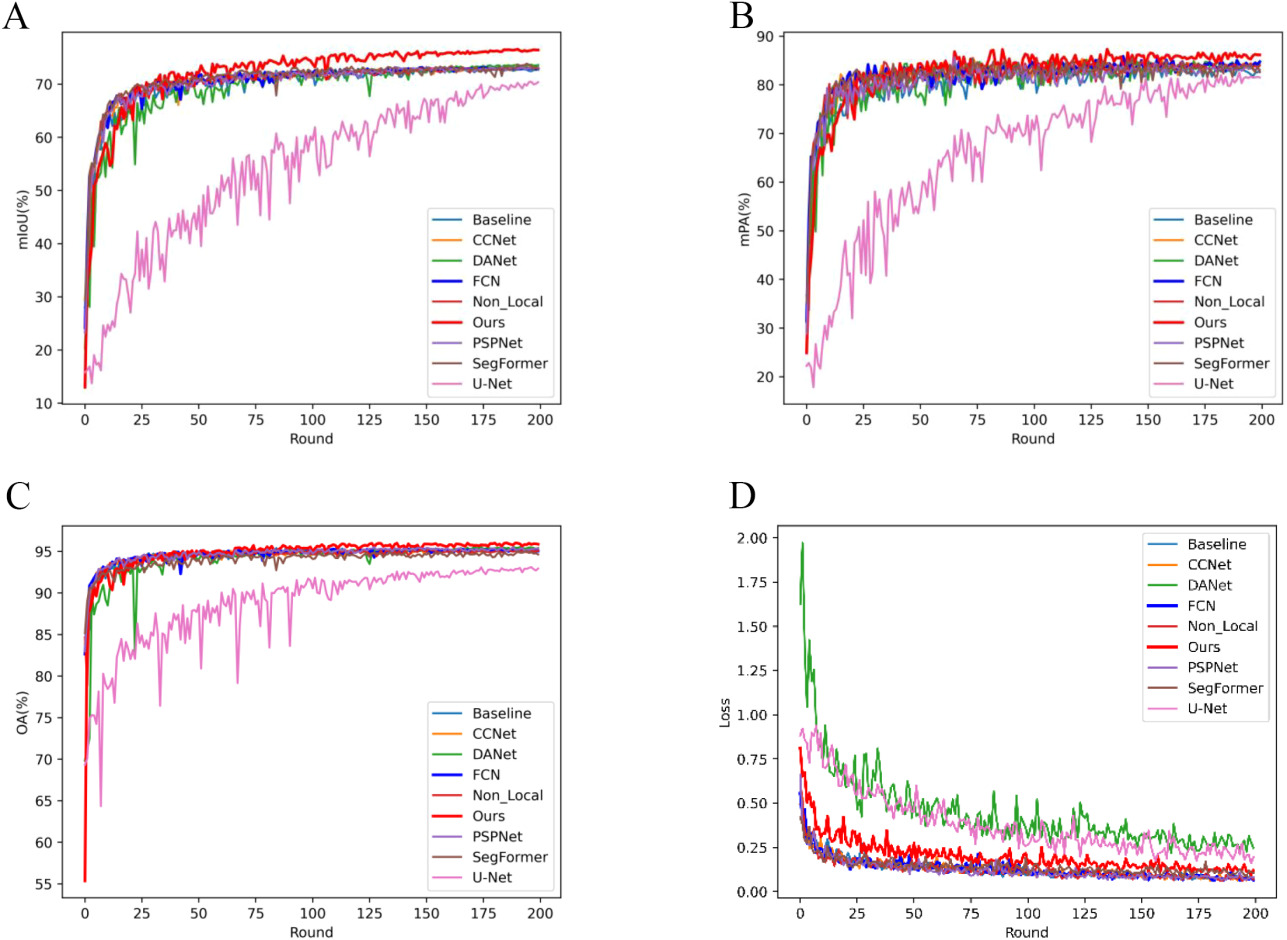
Evaluation Metrics and Loss Curve Analysis: This figure illustrates the progression of evaluation metrics and loss curves throughout the training process. Each Round consists of 50 iterations, with a total of 10,000 iterations completed.

**Figure 5** visualizes the performance trends and stability across models. **Figure 5A** depicts the mIoU trends per 50 iterations, highlighting that LT-DeepLab reaches higher mIoU levels faster and maintains greater stability compared to others, surpassing other models’ mIoU at 2500 iterations versus their 10000 iterations. **Figure 5B** shows the mPA change curves, with our model achieving significantly higher mPA after 3750 iterations. **Figure 5C** compares the OA curves, demonstrating that our model’s curve is markedly more stable. Lastly, **Figure 5D** presents the loss variation curves; despite using the same cross-entropy loss function as other models, our network employs auxiliary loss, aiding in quicker convergence and resulting in a slightly higher initial loss value. These data further affirm the superior performance of our model.

**Figure 6:**
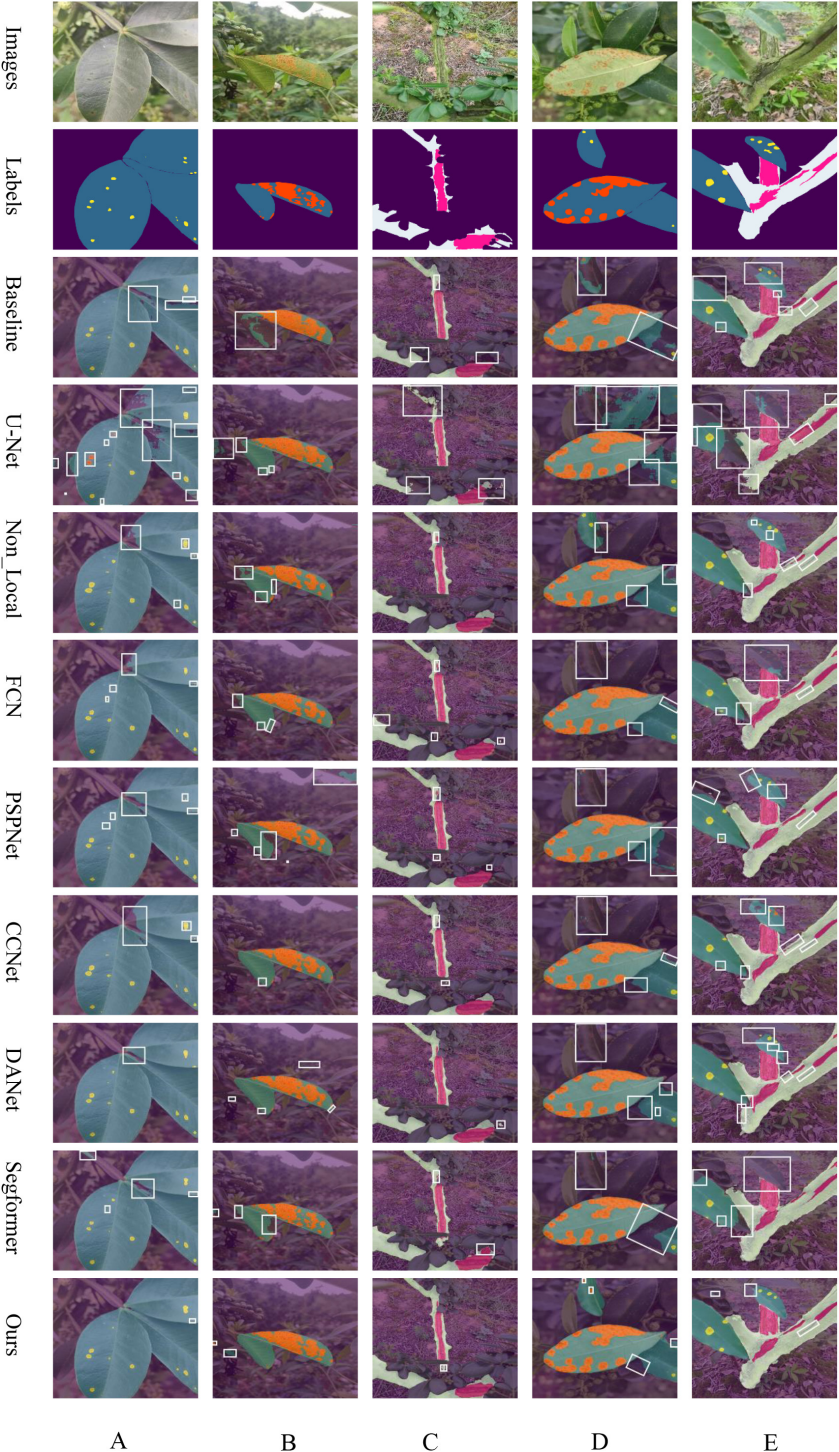
Overlay of Predicted Results from Common Networks: **(A)** leaf spot, **(B)** rust, **(C)** frost damage, **(D)** simultaneous rust and leaf spot, and **(E)** concurrent leaf spot and frost damage, respectively.

To demonstrate the distinct performance of various networks more effectively, this paper compares their prediction results as depicted in **Figure 6**. This comparison highlights the challenges posed by real-environment field conditions and varying scales. The baseline network utilizes the original ASPP module for feature extraction, which fails to adequately detail the leaf edges and poorly integrates areas where trunks meet leaves, indicating limited segmentation capability.

The UNet network, despite its success in healthcare applications, underperforms on our dataset. This is likely due to its smaller number of parameters and the U-shaped with skip connections, optimized for simpler semantic tasks. In contrast, the complexity of leaf and stem disease segmentation in Zanthoxylum bungeanum Maxim proves too challenging, resulting in suboptimal outcomes. Non_Local excels in capturing long-distance dependencies and uniquely succeeds in correctly segmenting distant diseased spots as seen in **Figure 6E**. However, it still struggles with accurate feature extraction at the edges of leaves, diseased spots, and trunk regions. FCN, a classic semantic segmentation network, retains spatial feature information effectively using a fully convolutional structure. It performs well in identifying larger targets within images but is unable to adequately segment smaller, less significant ones, often ignoring them completely. PSPNet, which incorporates a pyramid pooling module, manages boundary information more effectively than many networks by capturing contextual details at various scales. Yet, like FCN, it often overlook minor targets, needing further improvements in overall segmentation. CCNet, designed to reduce the computational intensity inherent in Non_Local through its Criss-Cross Attention mechanism, slightly outperforms Non_Local in segmenting target edges according to the comparative prediction images. DANet, which integrates both spatial and channel attention mechanisms, achieves the highest accuracy among the traditional CNN networks. Nonetheless, it still neglects elements in the distance. Our proposed LT-DeepLab network outshines all compared networks by delivering superior segmentation of target boundary information—such as leaf-to-lesion, leaf-to-background, trunk-to-leaf, and trunk-to-background transitions. It markedly surpasses other models, especially in segmenting very small leaf lesions, underscoring its superiority over common semantic segmentation networks.

**Figure 7:**
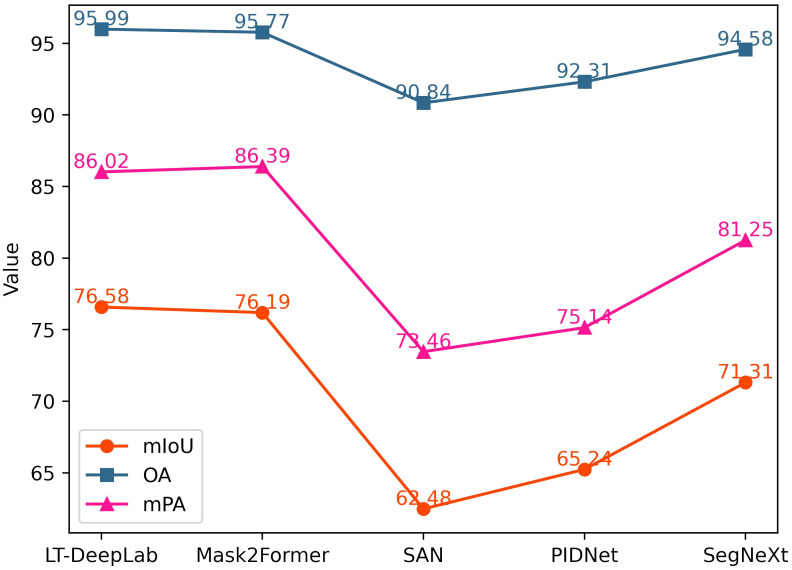
Frontier Model Comparison Line Chart.

In addition to the classical networks mentioned above, this paper also compares several recently proposed models, as shown in **Figure 7**. Mask2Former (Cheng et al., 2022) integrates the masking technique and self-attention mechanism into a fully convolutional network, achieving performance second only to our proposed LT-DeepLab, with an mIoU of 76.19%. SegNeXt (Guo et al., 2022) updates the design of the traditional convolutional block and utilizes multi-scale convolutional features to evoke spatial attention through simple elemental multiplication, achieving an mIoU of 71.31%. SAN (Xu et al., 2023) and PID (Xu et al., 2023), which focus more on lightweight design, perform poorly on our dataset, with mIoU values of 62.48% and 65.24%, respectively.

To demonstrate the effectiveness of the attention mechanism proposed in this paper, we replaced the CRCC attention mechanism in the LT-DeepLab model with various other attention mechanisms while keeping all other conditions constant. The results are presented in the **Table 2**. Using the Criss-Cross Attention (CCA) alone results in lower accuracy due to insufficient contextual connections of channel features. The CBAM alone achieves better results by focusing on both channel and spatial features. The CRCC module proposed in this paper, which enhances channel features using CBAM while retaining the spatial contextual linking capability of Criss-Cross Attention module, achieves the best results across all metrics. The ELA module (Xu and Wan, 2024) extracts feature vectors in the horizontal and vertical directions using band-pooling in the spatial dimension, resulting in mIoU, mPA, and OA values of 76.13%, 85.86%, and 95.74%, respectively. The CA module (Hou et al., 2021) employs global average pooling of feature maps in both the width and height directions, then merges the two parallel phases, achieving mIoU, mPA, and OA values of 76.22%, 85.33%, and 95.89%, respectively. The EMA module (Ouyang et al., 2023) reshapes some channels to obtain the batch dimension and groups them into multiple sub-features to preserve channel information, resulting in mIoU, mPA, and OA values of 76.01%, 85.41%, and 95.86%, respectively. The ECA module (Wang et al., 2020) captures inter-channel dependencies using one-dimensional convolution, avoiding the complex upscaling and downscaling process, with mIoU, mPA, and OA values of 76.04%, 85.41%, and 95.86%, respectively. These data further confirm the effectiveness and superiority of the attention mechanism proposed in this paper.

**Table 2:** Comparison of different attention mechanisms in LT-DeepLab.

Attention	mIoU	mPA	OA
CCA	75.81	86.02	95.77
CBAM	76.29	85.65	95.91
ELA	76.13	85.86	95.74
CA	76.22	85.33	95.89
EMA	76.01	85.86	95.80
ECA	76.04	85.41	95.86
CRCC (our)	**76.58**	**86.02**	**95.99**

Bold indicates that this metric has the best performance.

### 3.2 Heat map visualization

**Figure 8:**
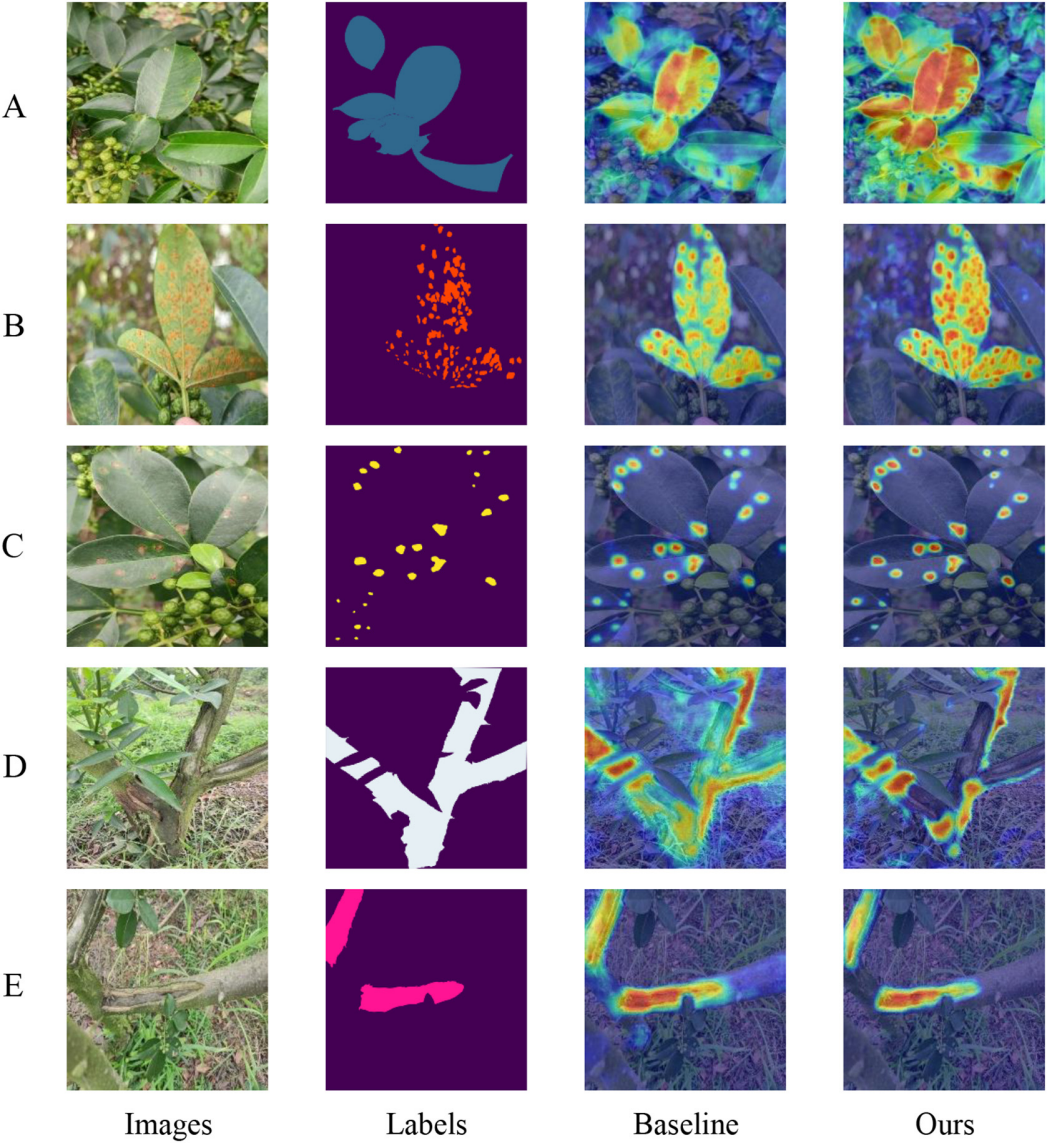
Category Weight Activation Maps: Panels A through E display the category weight activation maps for different conditions: **(A)** diseased leaves, **(B)** rust, **(C)** leaf spot, **(D)** diseased trunks, and **(E)** frost-damaged areas.

To visually demonstrate the enhancements in our network, this paper employes gradient-weighted class activation mapping (Grad-CAM) (Selvaraju et al., 2017) to illustrate how effectively the model discriminates between different classes. Grad-CAM is a technique that visualizes neural network decisions by analyzing gradients in the final convolutional layer to determine the significance of each feature map relative to a specific class. This method generates heat maps that highlight areas of the image most relevant to the model’s predictions. In **Figure 8**, this paper compares the heat maps from both the baseline and the improved versions of our model to showcase the differences pre- and post-enhancement. Each class is visualized separately to assess how effectively the network activates in response to that class. In the images, darker and more focused colors within the designated segmentation regions indicate stronger network activations, signifying better model performance and learning capability.

Upon comparison, it is evident that our model demonstrates superior category-specific activation compared to the baseline model. For instance, as shown in **Figure 8A**, our model is able to detect multiple diseased leaves against a complex background simultaneously, whereas the baseline model tends to recognize only few leaves. Similarly, in **Figure 8D**, while the baseline network struggles to accurately identify normal trunk sections, often misactivating diseased parts and some background areas, our model distinctly and correctly activates the normal trunk category. Overall, these observations confirm that our model achieves more precise class activation than the baseline model, validating the effectiveness of our enhanced attention mechanism.

### 3.3 Confusion matrix

**Figure 9:**
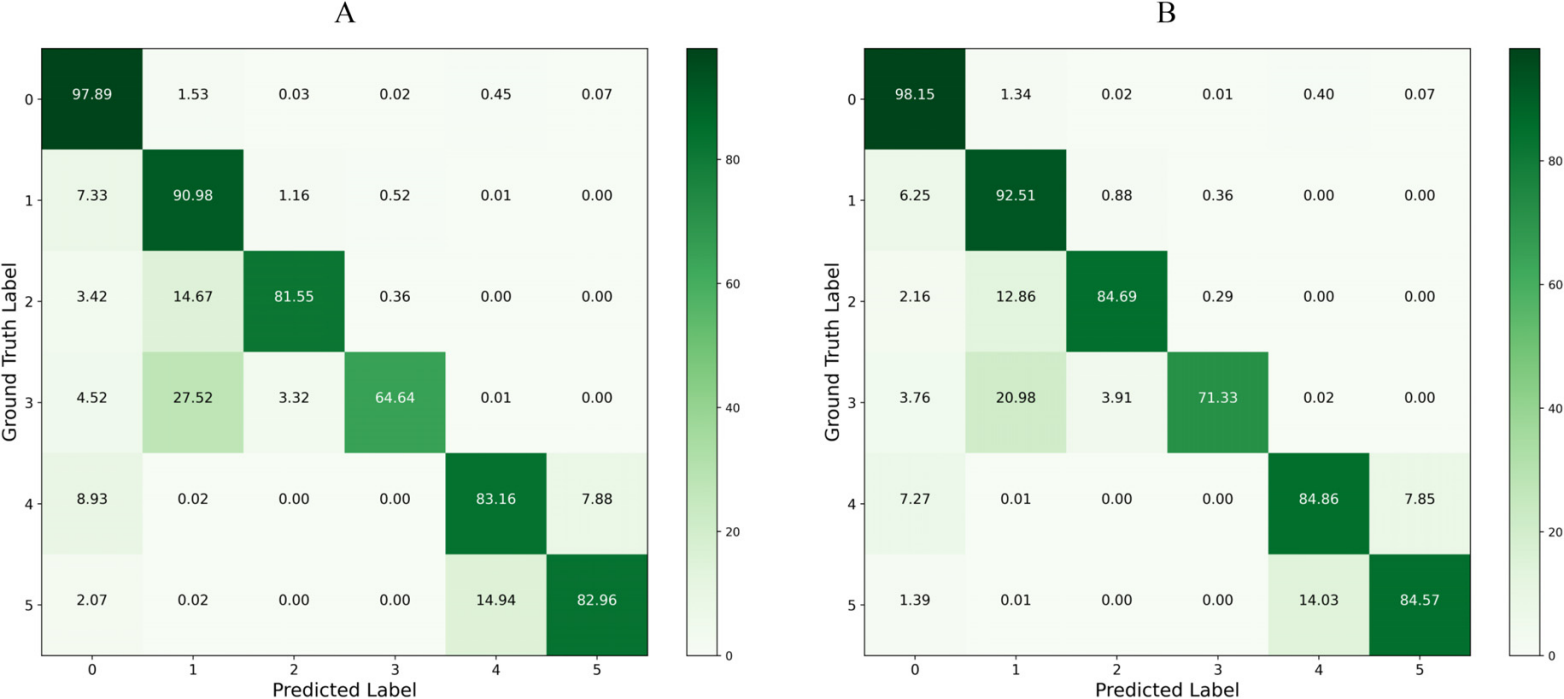
Confusion Matrix Comparison: Panel **(A)** displays the confusion matrix for the baseline model on the test set, and Panel **(B)** shows the confusion matrix for the LT-DeepLab model on the test set.

**Figures 9A, B** present the confusion matrices for the baseline model and our proposed model on the test set, respectively. By applying row normalization to the confusion matrices, the diagonal values in each matrix reflect the pixel accuracy of each category. A comparison of these values clearly demonstrates that our model achieves superior segmentation performance across all categories. For instance, an analysis of the first row shows that our model more effectively distinguishes between each category and the background, exhibiting significantly better performance in real-world environment segmentation compared to the baseline model.

### 3.4 Ablation experiments

To evaluate the effectiveness of our improved modules, this paper initially examines the impact of employing multiple auxiliary heads, as detailed in **Table 3**. Additionally, sixteen sets of ablation experiments are conducted to validate each of the four modules discussed in this paper, focusing on various metrics including model size and computational efficiency. These experiments are summarized in **Table 4**, which reports on metrics such as mIoU, mPA, OA, the floating-point operations (FLOPs), and the number of parameters (Params).The baseline configuration, native DeepLabv3+, utilizes a multilayer convolutional structure within the original ASPP, leading to a high computational demand of 0.27 TFLOPs and a parameter count of 65.74M, achieving an mIoU of 72.99%. As demonstrated in **Table 4** (2), the implementation of auxiliary loss results in a 0.15% improvement in mIoU. Importantly, since auxiliary loss only influences the training phase, it does not increase the number of parameters or computational load during the inference process. In experiment 3(3), integrating deformable convolution with low-dimensional feature maps resulted in a 1.47% increase in mIoU. **Table 4** (5) highlights the enhancements to the ASPP module through lightweight and attention-fused deformable convolutions, which not only elevate mIoU by 2.54% but also reduce the computational demand by 0.05 TFLOPs and decrease the parameter count by 13.37M. Further, as shown in **Table 4** (9), the inclusion of our improved CRCC module based on CCNet slightly raised the mIoU by 0.01%. A comprehensive comparison from **Tables 4** (8) and 3(16) demonstrates that the collective application of all improvements, with and without the CRCC module, boosts the mIoU by 0.46%, mPA by 0.01%, and OA by 0.28%, confirming the overall efficacy of our enhancements.

**Table 3:** Comparison of auxiliary head performance.

Name	mIoU	mPA	OA
DWFCNHead	76.28	86.07	95.95
DAHead	76.13	**86.33**	95.84
PSPHead	76.29	85.91	95.91
FCNHead	**76.58**	86.02	**95.99**

Bold indicates that this metric has the best performance.

**Table 4:** Comparison of evaluation indexes of ablation experiments with parametric quantities and calculation quantities.

Num	CRCC	FDCASPP	DCN	Aux_Loss	mIoU	mPA	OA	FLOPs/T	Params/M
1	✗	✗	✗	✗	72.99	83.53	95.36	0.27	65.74
2	✗	✗	✗	✓	73.14	84.03	95.34	0.27	65.74
3	✗	✗	✓	✗	74.46	84.26	95.52	0.29	67.55
4	✗	✗	✓	✓	74.25	84.05	95.48	0.29	67.55
5	✗	✓	✗	✗	75.53	85.15	95.69	**0.22**	**52.37**
6	✗	✓	✗	✓	75.56	85.44	95.70	**0.22**	**52.37**
7	✗	✓	✓	✗	76.14	85.63	95.66	0.23	53.08
8	✗	✓	✓	✓	76.12	86.01	95.71	0.23	53.08
9	✓	✗	✗	✗	73.00	83.13	95.35	0.28	67.34
10	✓	✗	✗	✓	73.29	83.52	95.46	0.28	67.34
11	✓	✗	✓	✗	74.60	84.54	95.14	0.29	69.15
12	✓	✗	✓	✓	74.55	84.97	95.71	0.29	69.15
13	✓	✓	✗	✗	75.43	85.11	95.75	0.23	54.68
14	✓	✓	✗	✓	75.61	85.43	95.78	0.23	54.68
15	✓	✓	✓	✗	76.03	85.40	95.88	0.24	54.68
16	✓	✓	✓	✓	**76.58**	**86.02**	**95.99**	0.24	54.68

In the table, a checkmark (✓) indicates that a specific module was included in that group of experiments, while a cross (✗) signifies that the module was not incorporated in that particular experimental setup.

Bold indicates that this metric has the best performance.

In pursuit of an optimal auxiliary head to further optimize training, this paper evaluates four different designs that generate auxiliary loss during training only. The results are summarized in **Table 3**, which led us to select the FCNHead as our auxiliary head due to its superior performance in mIoU and OA.

**Figure 10:**
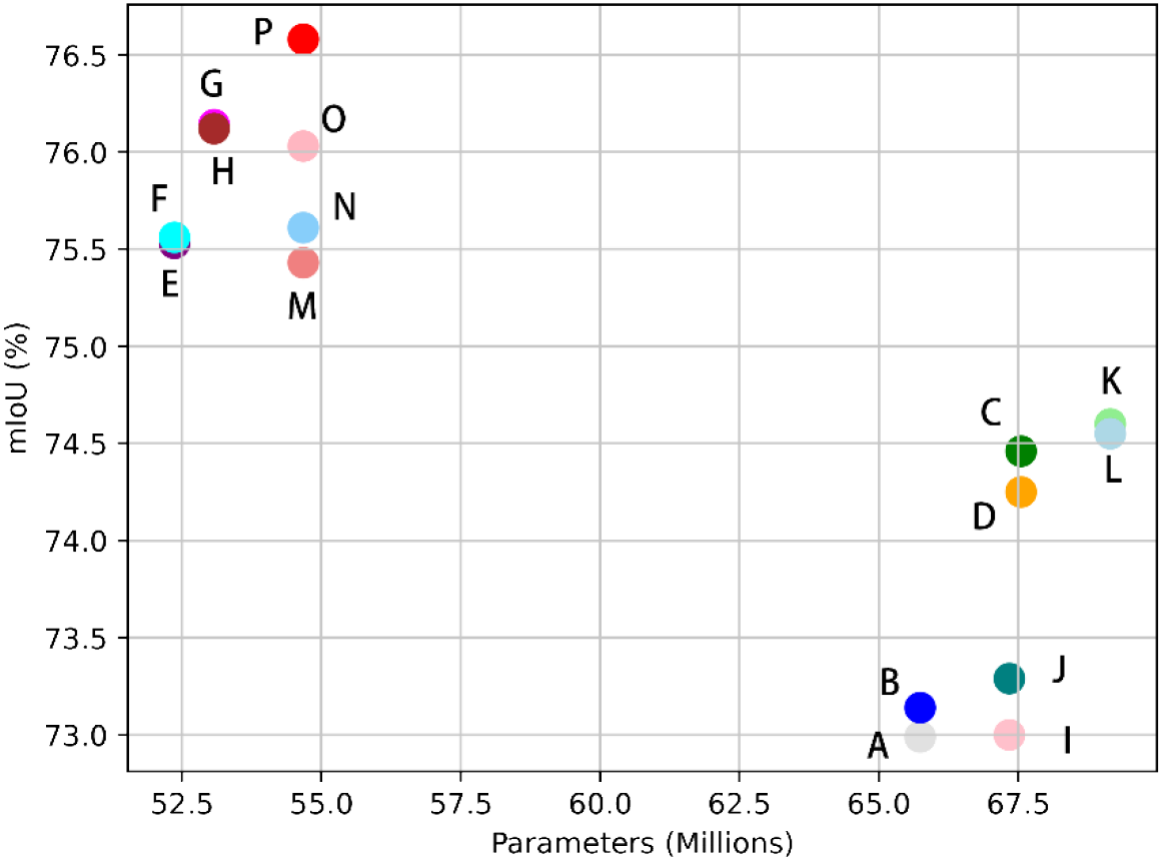
Scatter Plot of Evaluation Indicators versus Number of Parameters: Points A through P on the plot correspond to data entries 1 through 16 in **Table 4**, respectively.

**Figure 10** is a scatter Plot of mIoU versus Number of Parameters. This scatter plot illustrates the trade-off between model complexity and segmentation accuracy across the entire set of ablation experiments. It visualizes the relationship between the accuracy of each experimental group and their corresponding number of parameters. Notably, our model attains the highest mIoU while maintaining a relatively low parameter count, demonstrating its efficiency and effectiveness in segmentation tasks.

To validate the robustness of the LT-DeepLab network proposed in this paper, various backbone networks were employed for feature extraction. As depicted in **Table 5**, the experiments were divided into five groups. Each group compared the original DeepLabV3+ model with the corresponding backbone to the proposed LT-DeepLab model using the same backbone. Notably, in each group, the LT-DeepLab architecture consistently achieved the best segmentation performance across all metrics. The most significant improvement was observed in the first group, with an increase of 17.84% in mIoU, 18.14% in mPA, and 3.58% in OA. These enhancements can be attributed to the superior contextualization and feature integration capabilities of the LT-DeepLab network. The results clearly illustrate that the LT-DeepLab architecture is robust and versatile, making it a suitable choice for various feature extraction backbone networks.

**Table 5:** Comparison of different feature extraction backbone networks in LT-DeepLab.

Model	mIoU	mPA	OA
DeepLabV3Plus+replknet	53.75	64.96	90.75
LT-DeepLab+replknet	**71.59**	**83.10**	**94.33**
DeepLabV3Plus+vgg16	60.66	70.94	93.20
LT-DeepLab+vgg16	**73.49**	**83.91**	**95.30**
DeepLabV3Plus+MobileNetV3	56.79	67.30	92.19
LT-DeepLab+ MobileNetV3	**70.07**	**81.18**	**94.51**
DeepLabV3Plus+ResNeSt	69.76	80.28	94.56
LT-DeepLab+ ResNeSt	**72.59**	**83.03**	**95.06**
DeepLabV3Plus+ResNet(baseline)	72.99	83.53	95.36
LT-DeepLab+ResNet(our)	**76.58**	**86.02**	**95.99**

Bold indicates that this metric has the best performance.

**Figure 11:**
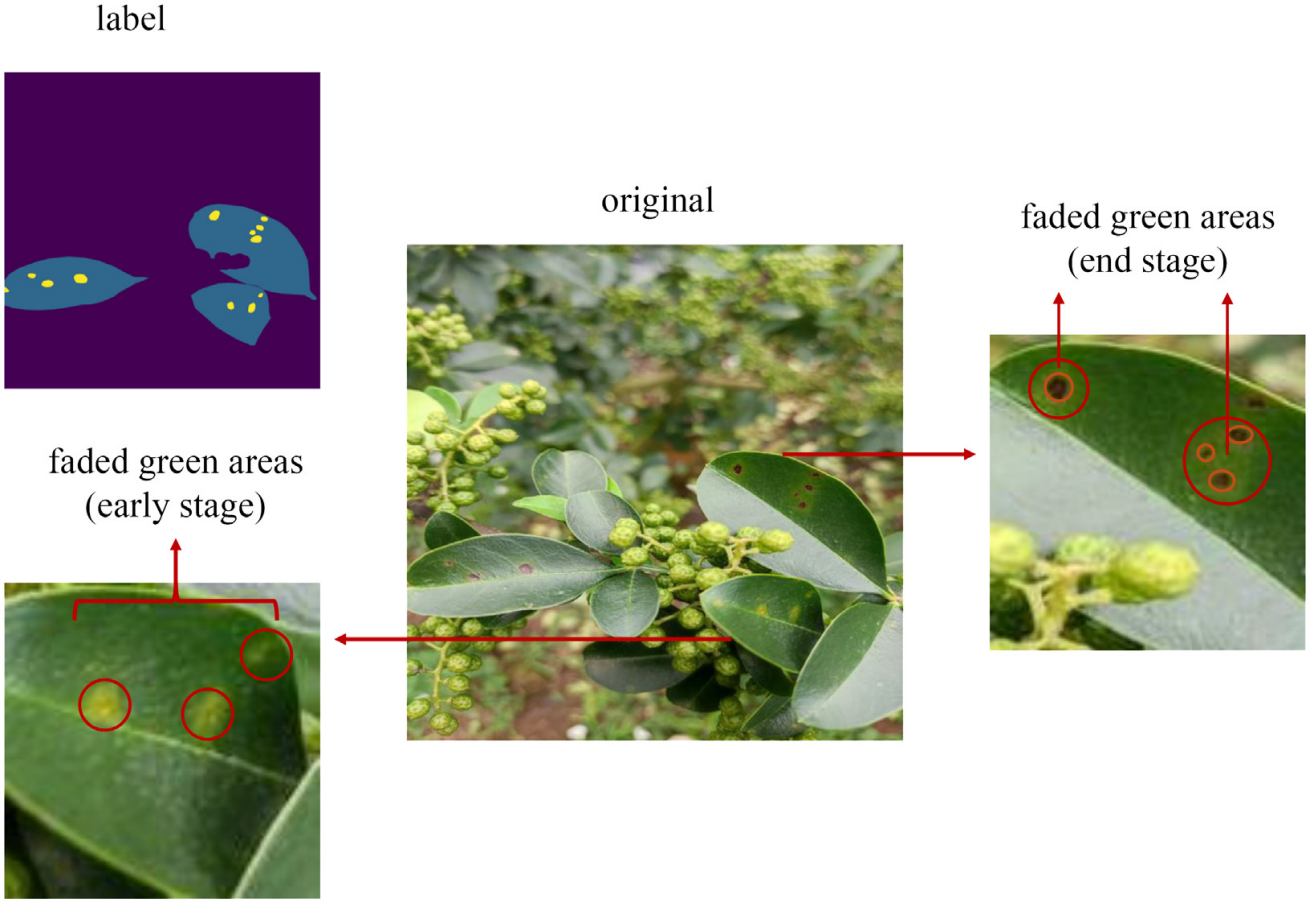
Detail of faded green areas.

**Table 6** presents the IoU values for each category in our proposed model, indicating that the IoU for leaf spot disease is the lowest. One probable reason for this is the small size and irregular boundaries of these spots. As depicted in **Figure 11**, these spots have a range of faded green areas around the brown spots, and in the early stages of the disease, only faded green spots are present without any brown spots. To ensure that the model can detect this type of disease even in its early stages, this paper includes the faded green areas in our labeling. However, the faintness of these boundaries results in less accurate information extraction, leading to lower segmentation accuracy for this category. Although this may reduce accuracy, it is crucial for identifying early-stage disease and enabling timely intervention to prevent further damage.

**Table 6:** Comparison of individual category IoUs between the baseline model and our model.

	0	1	2	3	4	5
Baseline	95.59	84.20	64.75	50.68	72.84	69.89
Ours	96.22	86.47	70.33	59.94	75.14	71.38

where 1-5 denote background, diseased leaves, rust, leaf spot, diseased trunks, frost damage respectively.

### 3.5 Module efficiency

To evaluate the computational efficiency of the primary modules proposed by LT-DeepLab, this study examines the number of parameters and computation time of the CRCC attention module and the FDCASPP structure. The results are presented in **Table 7**. The CRCC module is utilized in both the backbone feature extraction network and the FDCASPP structure, with the only difference being the number of intermediate channels. In the backbone, the CRCC module has 256 intermediate channels, whereas in the FDCASPP, it has 2048 intermediate channels. This design choice balances computational speed and segmentation accuracy. The average frame rate of the LT-DeepLab model during inference is 12.56 fps.

**Table 7:** Comparison of different feature extraction backbone networks in LT-DeepLab.

Module	Location	Params /M	-	Time/s	LT-DeepLab
CRCC	CRCC_backbone	1.1	Train_time	0.0273	12.56fps
			Test_time	0.0091	
	CRCC_FDCASPP	6.1	Train_time	0.0361	
			Test_time	0.0145	
FDCASPP	–	17.1	Train_time	0.0991	
			Test_time	0.0465	

The data dimensions of the aforementioned modules during training are (4, 256, 64, 64), while during testing, the dimensions are (1, 256, 86, 64). This discrepancy in feature map sizes between training and testing is attributable to the differing data augmentation strategies employed.

### 3.6 Significance test results

The results of the independent samples t-test indicate that the mean performance of the improved LT-DeepLab model is significantly higher than that of the baseline model. Specifically, the five experimental outcomes for LT-DeepLab were 76.48, 76.49, 76.49, 76.58, and 76.46, while the five outcomes for the baseline model were 72. 85, 72.88, 72.85, 72.99, and 72.82. The p-value obtained from the t-test was substantially lower than the commonly accepted significance level of 0.05, allowing us to reject the null hypothesis, thereby confirming that the mean difference between the two models is statistically significant. This finding demonstrates that LT-DeepLab significantly outperforms the baseline model in enhancing performance.

## 4 Discussion

In this study, we propose a novel CRCC attention mechanism integrated with DeepLabV3+, which simultaneously considers both spatial and channel-wise features. This mechanism skillfully combines the Criss-Cross attention with the CBAM mechanism, addressing the Criss-Cross attention’s limitation in capturing channel-wise features and enhancing overall performance. Additionally, we introduce a new cross-scale solution header, FCDASPP, which, compared to the original ASPP module, reduces the number of parameters by employing depthwise separable convolutions. This approach, when combined with the CRCC attention mechanism, significantly improves segmentation capabilities. Furthermore, the inclusion of an FCN auxiliary head enhances segmentation performance during training by participating solely in the loss computation, thereby avoiding any additional overhead during inference. The incorporation of deformable convolutions allows the convolutional kernels to learn offsets, facilitating the effective handling of shallow features and enabling more efficient extraction and fusion of shallow and deep features.

In the field of research on segmentation of leaf or trunk diseases in Zanthoxylum bungeanum Maxim, Yang et al. (2021) introduced a fifth ASPP branch into DeepLabv2 to segment rust disease in a controlled laboratory environment, achieving an mIoU of 84.99%. Zhang et al. (2024) proposed a lightweight U-shaped perceptual transformer for grape leaf disease segmentation, which strikes a balance between performance and efficiency. However, this method may not be suitable for field conditions and is limited to a small number of diseases, indicating that its practical applicability needs improvement. Wang et al. (2021) employed a two-stage segmentation approach for cucumber leaf disease in complex environments, using two networks sequentially to segment different targets, thereby achieving higher segmentation accuracy. However, this method incurs significant computational overhead, making it less practical for real-world production applications. The model proposed in this paper maintains accuracy while reducing the size compared to the benchmark network, enabling robust segmentation of multiple diseases simultaneously. Similar to the study by (Zhou et al., 2024), we also chose to improve the DeepLabV3+ model. The difference lies in their introduction of a gated pyramid feature fusion structure, which connects features of different scales using a specialized gating mechanism while capturing different receptive fields. The FDCASPP structure proposed in this paper is fundamentally designed to fuse multi-scale features and enhance the connections between them through the CRCC attention mechanism. Furthermore, Wang et al. (2024) demonstrated that integrating the CBAM attention mechanism with residuals into the UNet network enhances its feature extraction capability and ability to capture fine-grained information, utilizing an improved ASPP module. Our study extends this idea by complementing CBAM with the Criss-Cross attention mechanism, incorporating this combination into the FDCASPP structure to further enhance multi-scale feature extraction. Zhu et al. (2024) replaced dilated convolutions in the ASPP module with deformable convolutions to address issues such as poor segmentation accuracy for irregular defects in navel orange surface defect detection. Similarly, this study employs deformable convolutions in the self-proposed FDCASPP structure to assist in feature extraction, particularly in the decoder stage, to better fit irregular lesions on leaves and trunks, further demonstrating the effectiveness of deformable convolutions in extracting features from irregular targets. However, when comparing our study with the UNet network, unlike (Han et al., 2024) success in brain tumor image segmentation by integrating the Criss-Cross attention mechanism into UNet, the U-Net network performed poorly on our dataset. This could be attributed to UNet’s skip connection structure, which directly combines low-level and high-level features, leading to issues such as unclear boundaries when dealing with targets that have fuzzy edges or are difficult to distinguish from the background. In our dataset, diseased leaves are particularly challenging to differentiate from healthy ones in a complex environment, with the boundaries of the lesions being especially indistinct.

The model proposed in this paper strikes a balance between accuracy and model size when compared to the baseline network. It also demonstrates strong robustness by effectively segmenting multiple diseases simultaneously. Future work will focus on validating the model’s generalizability using other datasets. Despite the current model’s success in reducing computational load and parameter count, there remains room for further optimization. Future research will aim to develop a lighter and more efficient network, facilitating easier deployment in field environments. Additionally, the possibility of segmenting peppercorn fruits will be explored, enabling real-time monitoring and segmentation of Zanthoxylum bungeanum Maxim diseases to enhance the efficiency and productivity of peppercorn cultivation.

## 5 Conclusion

To address the challenge of integrated segmentation of diseases on Zanthoxylum bungeanum Maxim leaves and trunks, this research proposes an enhanced version of DeepLabv3+, named LT-DeepLab. This method innovatively applies semantic segmentation technology for joint disease targeting on both leaves and trunks. This paper have improved the Criss-Cross Attention module by integrating the channel-space attention capabilities of the CBAM, and proposed a new attention mechanism, the CRCC module, which accurately extracts edge information of leaves and trunks. Additionally, a deformable convolution module has been implemented to effectively capture low-dimensional information, enhancing the fusion with high-dimensional feature maps. Addressing the issue of the original ASPP module’s high parameter count and limited cross-scale information extraction capability, this paper has developed the FDCASPP module, designed to enhance the extraction of multi-scale information and improve target segmentation in complex backgrounds. Experimental results demonstrate that LT-DeepLab’s segmentation capabilities in complex environments surpass those of other commonly used semantic segmentation networks. Relative to the baseline model, LT-DeepLab not only reduces the number of parameters and computational demands but also achieves superior performance across all evaluation metrics.

## Data Availability

The raw data supporting the conclusions of this article will be made available by the authors, without undue reservation.

## References

[B1] Attri I. Awasthi L. K. Sharma T. P. Rathee P. (2023). A review of deep learning techniques used in agriculture. Ecol. Inf. 77, 102217. doi: 10.1016/j.ecoinf.2023.102217

[B2] Barbedo J. (2016). A novel algorithm for semi-automatic segmentation of plant leaf disease symptoms using digital image processing. Trop. Plant Pathol. 41, 210–224. doi: 10.1007/s40858-016-0090-8

[B3] Chen L.-C. Zhu Y. Papandreou G. Schroff F. Adam H. (2018). “Encoder-decoder with atrous separable convolution for semantic image segmentation,” in 15th European Conference on Computer Vision, Berlin, Springer.

[B4] Cheng B. Misra I. Schwing A. G. Kirillov A. Girdhar R. (2022). “Masked-attention mask transformer for universal image segmentation,” in 2022 IEEE/CVF Conference on Computer Vision and Pattern Recognition (CVPR), Piscataway, NJ: IEEE.

[B5] Cruz A. Ampatzidis Y. Pierro R. Materazzi A. Panattoni A. De Bellis L.. (2019). Detection of grapevine yellows symptoms in Vitis vinifera L. with artificial intelligence. Comput. Electron. Agric. 157, 63–76. doi: 10.1016/j.compag.2018.12.028

[B6] Dai J. Qi H. Xiong Y. Li Y. Zhang G. Hu H.. (2017). “Deformable convolutional networks,” in 2017 IEEE International Conference on Computer Vision (ICCV), Piscataway, NJ: IEEE.

[B7] Deng Y. Xi H. Zhou G. Chen A. Wang Y. Li L.. (2023). An effective image-based tomato leaf disease segmentation method using MC-UNet. Plant Phenomics 5, 0049. doi: 10.34133/plantphenomics.0049 37228512 PMC10204749

[B8] Gao L. Lin X. (2019). Fully automatic segmentation method for medicinal plant leaf images in complex background. Comput. Electron. Agric. 164, 104924. doi: 10.1016/j.compag.2019.104924

[B9] Guo M.-H. Lu C.-Z. Hou Q. Liu Z. Cheng M.-M. Hu S.-M. (2022). Segnext: Rethinking convolutional attention design for semantic segmentation. Adv. Neural Inf. Process. Syst. 35, 1140–1156.

[B10] Han X. Liu J. Zhao J. (2024). “U-CCNet: brain tumor MRI image segmentation model with broader global context semantic information abstraction,” in 2024 IEEE 7th Advanced Information Technology, Electronic and Automation Control Conference (IAEAC). (Piscataway, NJ: IEEE), 1550–1554.

[B11] Hou Q. Zhou D. Feng J. (2021). “Coordinate attention for efficient mobile network design,” in 2021 IEEE/CVF Conference on Computer Vision and Pattern Recognition (CVPR), Piscataway, NJ: IEEE.

[B12] Huang Z. Wang X. Huang L. Huang C. Wei Y. Liu W. (2019). “Ccnet: Criss-cross attention for semantic segmentation,” in 2019 IEEE/CVF International Conference on Computer Vision (ICCV), Piscataway, NJ: IEEE.

[B13] Javidan S. M. Banakar A. Vakilian K. A. Ampatzidis Y. (2023). Diagnosis of grape leaf diseases using automatic K-means clustering and machine learning. Smart Agric. Technol. 3, 100081. doi: 10.1016/j.atech.2022.100081

[B14] Jodas D. S. Brazolin S. Yojo T. De Lima R. A. Velasco G. D. N. MaChado A. R.. (2021). “A deep learning-based approach for tree trunk segmentation,” in 2021 34th SIBGRAPI Conference on Graphics, Patterns and Images (SIBGRAPI), Piscataway, NJ: IEEE.

[B15] Li D. Cao Y. Tang X.-s. Yan S. Cai X. (2018). Leaf segmentation on dense plant point clouds with facet region growing. Sensors 18, 3625. doi: 10.3390/s18113625 30366434 PMC6263610

[B16] Liu Y. Yu Q. Geng S. (2024). Real-time and lightweight detection of grape diseases based on Fusion Transformer YOLO. Front. Plant Sci. 15, 1269423. doi: 10.3389/fpls.2024.1269423 38463562 PMC10920279

[B17] Lu J. Xiang J. Liu T. Gao Z. Liao M. (2022). Sichuan pepper recognition in complex environments: A comparison study of traditional segmentation versus deep learning methods. Agriculture 12, 1631. doi: 10.3390/agriculture12101631

[B18] Ma J. Du K. Zhang L. Zheng F. Chu J. Sun Z. (2017). A segmentation method for greenhouse vegetable foliar disease spots images using color information and region growing. Comput. Electron. Agric. 142, 110–117. doi: 10.1016/j.compag.2017.08.023

[B19] Mzoughi O. Yahiaoui I. (2023). Deep learning-based segmentation for disease identification. Ecol. Inf. 75, 102000. doi: 10.1016/j.ecoinf.2023.102000

[B20] Nahiduzzaman M. Chowdhury M. E. Salam A. Nahid E. Ahmed F. Al-Emadi N.. (2023). Explainable deep learning model for automatic mulberry leaf disease classification. Front. Plant Sci. 14, 1175515. doi: 10.3389/fpls.2023.1175515 37794930 PMC10546311

[B21] Ouyang D. He S. Zhang G. Luo M. Guo H. Zhan J.. (2023). “Efficient multi-scale attention module with cross-spatial learning,” in 2023 IEEE International Conference on Acoustics, Speech and Signal Processing (ICASSP), Piscataway, NJ: IEEE.

[B22] Pal A. Kumar V. (2023). AgriDet: Plant Leaf Disease severity classification using agriculture detection framework. Eng. Appl. Artif. Intell. 119, 105754. doi: 10.1016/j.engappai.2022.105754

[B23] Qi M. Jia G. (2023). “Infrared small target detection algorithm based on improved deepLabV3+,” in 2023 8th International Conference on Intelligent Computing and Signal Processing (ICSP), Piscataway, NJ: IEEE.

[B24] Savary S. Willocquet L. Pethybridge S. J. Esker P. McRoberts N. Nelson A. (2019). The global burden of pathogens and pests on major food crops. Nat. Ecol. Evol. 3, 430–439. doi: 10.1038/s41559-018-0793-y 30718852

[B25] Selvaraju R. R. Cogswell M. Das A. Vedantam R. Parikh D. Batra D. (2017). “Grad-cam: Visual explanations from deep networks via gradient-based localization,” in 2017 IEEE International Conference on Computer Vision (ICCV), Piscataway, NJ: IEEE.

[B26] Shedthi B. ,. S. Siddappa M. Shetty S. Shetty V. Suresh R. (2023). Detection and classification of diseased plant leaf images using hybrid algorithm. Multimedia Tools Appl. 82, 32349–32372. doi: 10.1007/s11042-023-14751-0

[B27] Sifre L. Mallat S. (2014). Rigid-motion scattering for texture classification. arXiv. arXiv:1403.1687. doi: 10.48550/arXiv.1403.1687

[B28] Thai H.-T. Le K.-H. Nguyen N. L.-T. (2023). FormerLeaf: An efficient vision transformer for Cassava Leaf Disease detection. Comput. Electron. Agric. 204, 107518. doi: 10.1016/j.compag.2022.107518

[B29] Wang C. Du P. Wu H. Li J. Zhao C. Zhu H. (2021). A cucumber leaf disease severity classification method based on the fusion of DeepLabV3+ and U-Net. Comput. Electron. Agric. 189, 106373. doi: 10.1016/j.compag.2021.106373

[B30] Wang J. Jia J. Zhang Y. Wang H. Zhu S. (2024). RAAWC-UNet: an apple leaf and disease segmentation method based on residual attention and atrous spatial pyramid pooling improved UNet with weight compression loss. Front. Plant Sci. 15, 1305358. doi: 10.3389/fpls.2024.1305358 38529067 PMC10961398

[B31] Wang Q. Wu B. Zhu P. Li P. Zuo W. Hu Q. (2020). “ECA-Net: Efficient channel attention for deep convolutional neural networks,” in 2020 IEEE/CVF Conference on Computer Vision and Pattern Recognition (CVPR), Piscataway, NJ: IEEE.

[B32] Woo S. Park J. Lee J.-Y. Kweon I. S. (2018). “Cbam: Convolutional block attention module,” in 15th European Conference on Computer Vision, Berlin: Springer.

[B33] Xu B. Wang N. Chen T. Li M. (2015). Empirical evaluation of rectified activations in convolutional network. arXiv preprint. arXiv:1505.00853. doi: 10.48550/arXiv.1505.00853

[B34] Xu J. Xiong Z. Bhattacharyya S. P. (2023). “PIDNet: A real-time semantic segmentation network inspired by PID controllers,” in 2023 IEEE/CVF Conference on Computer Vision and Pattern Recognition (CVPR), Piscataway, NJ: IEEE.

[B35] Xu M. Zhang Z. Wei F. Hu H. Bai X. (2023). “Side adapter network for open-vocabulary semantic segmentation,” in 2023 IEEE/CVF Conference on Computer Vision and Pattern Recognition (CVPR), Piscataway, NJ: IEEE. 10.1109/TPAMI.2023.3311618 37665708

[B36] Xu W. Wan Y. (2024). ELA: efficient local attention for deep convolutional neural networks. arXiv preprint. arXiv:2403.01123. doi: 10.48550/arXiv.2403.01123

[B37] Yang F. Xu J. Wei H. Ye M. Xu M. Fu Q.. (2021). Multi-scale image segmentation model for fine-grained recognition of Zanthoxylum rust. Computers, Materials and Continua 71, 2963–2980. doi: 10.32604/cmc.2022.022810

[B38] Zhang X. Li F. Jin H. Mu W. (2023). Local Reversible Transformer for semantic segmentation of grape leaf diseases. Appl. Soft Computing 143, 110392. doi: 10.1016/j.asoc.2023.110392

[B39] Zhang X. Li F. Zheng H. Mu W. (2024). UPFormer: U-shaped perception lightweight transformer for segmentation of field grape leaf diseases. Expert Syst. Appl. 249, 123546. doi: 10.1016/j.eswa.2024.123546

[B40] Zhao H. Shi J. Qi X. Wang X. Jia J. (2017). “Pyramid scene parsing network,” in 2017 IEEE/CVF Conference on Computer Vision and Pattern Recognition (CVPR), Piscataway, NJ: IEEE.

[B41] Zhou H. Peng Y. Zhang R. He Y. Li L. Xiao W. (2024). GS-deepLabV3+: A mountain tea disease segmentation network based on improved shuffle attention and gated multidimensional feature extraction. Crop Prot. 183, 106762. doi: 10.1016/j.cropro.2024.106762

[B42] Zhu Y. Liu S. Wu X. Gao L. Xu Y. (2024). Multi-class segmentation of navel orange surface defects based on improved deeplabv3+. J. Agric. Eng. 55, 263–275. doi: 10.4081/jae.2024.1564

[B43] Zhu S. Ma W. Lu J. Ren B. Wang C. Wang J. (2023). A novel approach for apple leaf disease image segmentation in complex scenes based on two-stage DeepLabv3+ with adaptive loss. Comput. Electron. Agric. 204, 107539. doi: 10.1016/j.compag.2022.107539

